# A Perfect Storm: Ventricular Fibrillation Cardiac Arrest Due to Acute Myocardial Infarction Seen in the Postpartum Period in the Setting of COVID

**DOI:** 10.7759/cureus.40782

**Published:** 2023-06-22

**Authors:** Abdul Haseeb Riaz, Alison Gordon, Manoj Bhandari

**Affiliations:** 1 Internal Medicine, Campbell University, Cape Fear Valley Medical Center, Fayetteville, USA; 2 Medicine, Campbell University, School of Osteopathic Medicine, Fayetteville, USA; 3 Cardiology, Cape Fear Valley Medical Center, Fayetteville, USA

**Keywords:** cardiac arrest, vfib, covid, cardiomyopathy, post partum

## Abstract

Acute myocardial infarction (AMI) in women of reproductive age is uncommon; however, the risk of AMI increases four to five-fold during pregnancy as compared to non-pregnant women of similar age. In the period following childbirth, the incidence of AMI is often attributed to a range of factors. These factors encompass coronary vessel dissection related to atherosclerosis, thrombosis, coronary spasm, and, in rare cases, takotsubo cardiomyopathy. The physiological alterations that accompany pregnancy induce a state of heightened coagulation, thereby elevating the risk of ischemic heart disease (IHD) in women. While the presence of traditional risk factors is not a strong predictor of post-partum AMI (PAMI) due to any cause, most cases of PAMI due to IHD or atherosclerosis have other mechanisms associated with AMI, significant past medical history, or the presence of other traditional risk factors. The purpose of this report is to describe an uncommon case of pregnancy associated with MI due to ischemic heart disease and discuss the pathogenesis of contributing risk factors that created the “perfect storm” leading to her presentation.

## Introduction

During pregnancy, the body increases production and concentrations of coagulation factors (VII, VIII, X, von Willebrand factor, and fibrinogen) to prepare for the increased risk of bleeding associated with miscarriage and childbirth to protect from severe hemorrhage [1-3]. There is also an increase in platelet aggregation due to endothelial stress [4]. These physiological changes have been seen to persist post-partum and are associated with hypercoagulability and the increased risk of thrombosis and thromboembolism [5]. The presence of traditional risk factors is not a strong predictor of post-partum acute myocardial infarction (PAMI) due to any cause; however, most cases of PAMI due to ischemic heart disease (IHD) or related to this hypercoagulable state have the presence of other mechanisms associated with acute myocardial infarction (AMI), significant past medical history, or other traditional risk factors [1,5].

Literature on IHD in pregnancy or during the postpartum period is scarce, likely due to this being a less common cause of PAMI with limited published cases. Factors that have been identified to increase the risk of thrombosis in postpartum women include a history of thrombosis, decreased physical activity later in pregnancy, thrombophilia, advanced maternal age, ethnicity, and obesity, among others [5]. Other cardiovascular risk factors associated with an increased risk of disease not specific to pregnancy include tobacco abuse, certain medical conditions, obesity, hyperlipidemia, or other causes of hypercoagulable states that can cause thrombosis, including coronavirus disease 2019 (COVID-19) can place patients at an even greater risk [1].

The patient seen in this case had multiple risk factors, creating a “perfect storm”, which likely contributed to the acute presentation of ST-elevation myocardial infarction (MI) due to thrombotic occlusion of a coronary vessel.

## Case presentation

**Figure 1:**
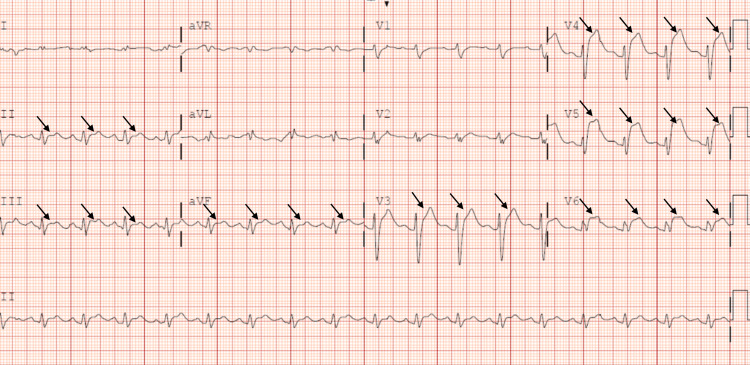
EKG showing ST-segment elevations in leads II, III, aVF, and V3-V6 (indicated by black arrows)

A two-month postpartum 36-year-old G3P3003 female with a past medical history significant for hypertension, tobacco use, obesity (BMI 32.9), no personal or family history of hypercoagulable disorders, and no family history of coronary artery disease presented to the emergency department via emergency medical services (EMS) in ventricular fibrillation (V-Fib) cardiac arrest. History revealed that the patient had been experiencing intermittent, substernal sharp chest pain since the day prior, which ultimately worsened in intensity with radiation to the left arm and jaw, prompting her to call 911. When EMS first arrived on the scene, the patient was awake and responsive but had cardiopulmonary arrest shortly after. Cardiopulmonary resuscitation (CPR) was initiated and the rhythm identified was V-Fib. The patient received three rounds of CPR with EMS along with three defibrillatory shocks and administration of epinephrine and amiodarone per the advanced cardiac life support (ACLS) protocol. Upon arrival at the emergency department, the patient was found to be obtunded, with a Glasgow Coma Scale (GCS) of 3. CPR was continued and rhythm was again identified to be V-Fib. She required defibrillation two additional times with a subsequent return of spontaneous circulation (ROSC) and a total downtime of 10 minutes. The patient was then intubated and placed on mechanical ventilation. Initial lab work was remarkable for hypokalemia with potassium of 3, acute kidney injury (AKI) with a creatinine of 1.25, mild leukocytosis of 13.1, and initial troponin I of 0.114, which quickly rose to 126 on subsequent recheck. EKG post-ROSC was concerning for ST-elevation MI with ST-segment elevations observed in leads II, III, aVF, and V3-V6 (Figure 1). 

**Figure 2:**
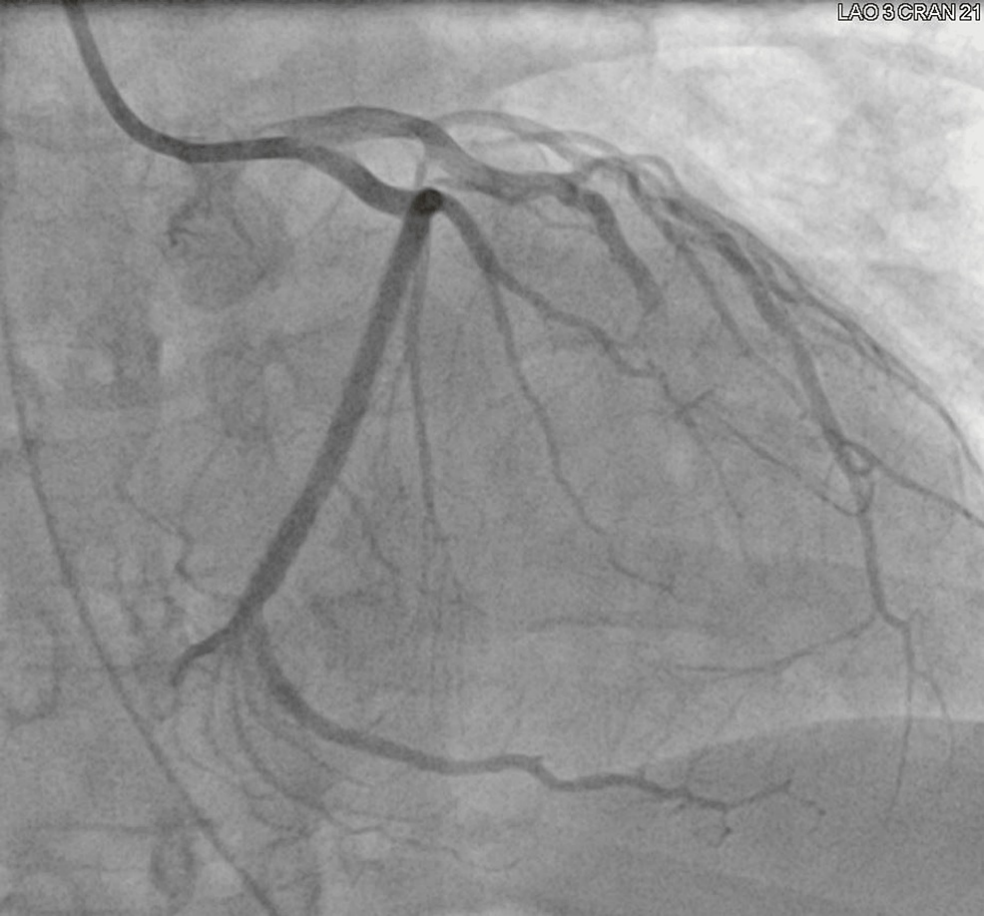
Coronary angiogram showing complete LAD occlusion LAD: Left Anterior Descending Artery

**Figure 3:**
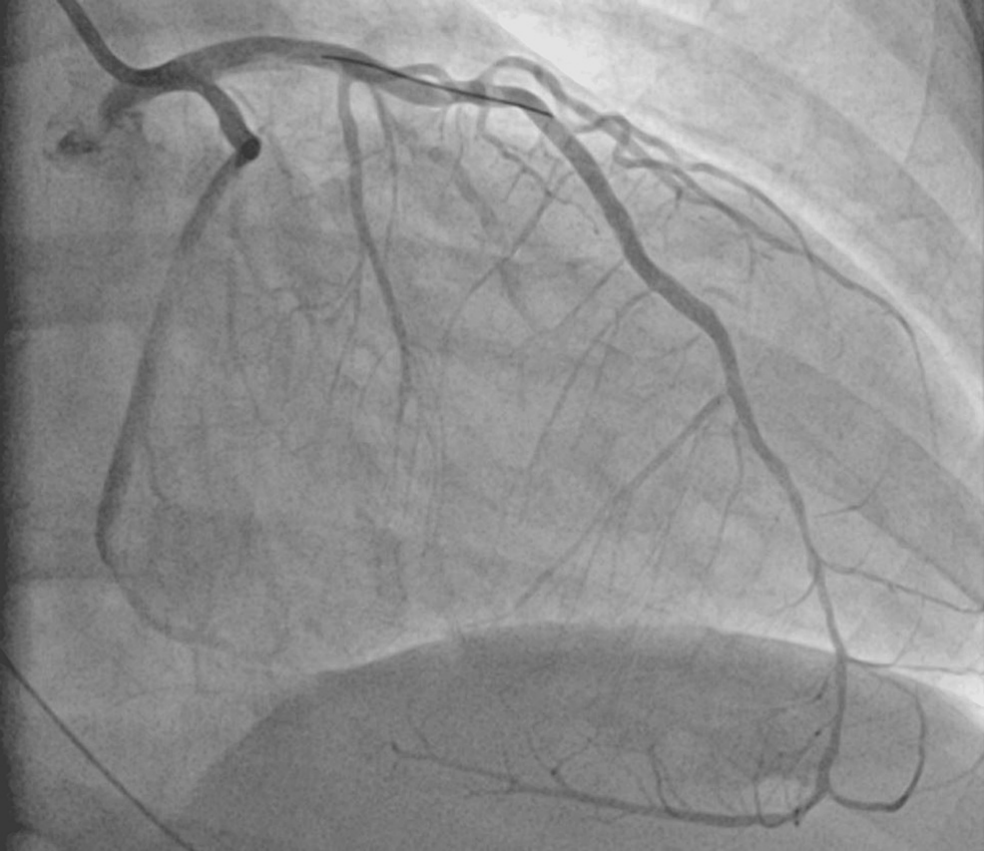
Coronary angiogram post-intervention with no residual stenosis of the LAD LAD: Left Anterior Descending Artery

An emergent coronary angiogram revealed significant single-vessel coronary artery disease with complete thrombotic occlusion of a large “wrap-around” left anterior descending artery (LAD) with no significant vessel dissection noted (Figure 2). Balloon angioplasty followed by drug-eluting stenting was performed for which there was an excellent post-interventional angiographic appearance with 0% residual stenosis (Figure 3). Ventriculography (LV Gram) revealed severely reduced left ventricular ejection fraction (LVEF) (25-30%) and findings consistent with a large anteroapical infarct.

Following primary percutaneous coronary intervention (PCI), she was then transferred to the ICU and started on aspirin and Brilinta. The patient was given a bolus of bivalrudin during PCI and a four-hour bivalrudin infusion was continued for anticoagulation per guidelines. While in the ICU, she was placed on vasopressors for post-cardiac arrest shock as indicated by low blood pressure and tachycardia. The patient continued to improve during her ICU stay and was eventually weaned off sedation, extubated, and placed on a nasal cannula. Initiation of post-PCI medications, including beta blockers and angiotensin-converting enzyme (ACE) inhibitors was delayed due to low blood pressure but were eventually started during her admission. Her COVID-19 polymerase chain reaction (PCR) results were positive. Chest X-ray was obtained showing minimal densities with no evidence of focal consolidations. Infectious disease was consulted, and the COVID treatment protocol was not initiated due to her acute cardiac event. She remained on supplemental oxygen for the duration of her hospitalization although did not develop any further complications in regards to COVID infection.

Follow-up echocardiogram prior to discharge showed similar findings to a prior LV Gram performed during angiogram with significant systolic dysfunction and LVEF 35%. These findings of acute systolic heart failure were most consistent with acute MI and cardiac arrest versus postpartum cardiomyopathy as there was a clearly identifiable cause. Risk factors in this case of thrombotic occlusion causing acute ST-elevation myocardial infarction (STEMI) include hypercoagulability in the setting of the post-partum period and COVID as well as a history of smoking as an independent risk factor.

This patient was eventually discharged home and completed outpatient cardiac rehab with close cardiology follow-up. A follow-up echocardiogram revealed complete recovery of LV function. The patient continues to do well and has since quit smoking.

## Discussion

Pregnancy-associated ischemic heart disease is an uncommon cause of acute MI seen in young women of reproductive age. The pathogenesis of coronary vessel occlusion as described in this case was most likely due to the underlying hypercoagulable state associated with the physiological changes of pregnancy and the presence of other risk factors of thrombosis and cardiovascular disease.

Pregnancy is associated with normal physiologic changes that occur to prepare and protect mom and baby through the duration of pregnancy and the postpartum period. During pregnancy, the body increases production and concentrations of coagulation factors (VII, VIII, X, von Willebrand factor, and fibrinogen) to prepare for the risk of bleeding associated with miscarriage and childbirth in order to protect the mother from severe hemorrhage [6,7]. The increase in coagulation factors while physiologically protective also creates a hypercoagulable state and increases the risk of maladaptive clot formation. In addition to hypercoagulability, physiological changes including vascular injury, endothelial stress, and hormone-induced vascular changes are important factors to be considered in the development of thrombosis and pathogenesis of thrombotic occlusion of coronary arteries [5,7]. The risk of thrombosis is higher postpartum than during pregnancy likely due to an imbalance of persistence of the described physiological changes and resolution of other changes that favor bleeding. During pregnancy, platelet counts are decreased, otherwise known as gestational thrombocytopenia, which balances out the hypercoagulability as described [5]. Shortly after delivery, gestational thrombocytopenia spontaneously resolves while the other factors that work to increase coagulability remain thus making the risk of thrombosis greater in the postpartum period [6]. This underlying, physiological risk for pregnancy-associated thrombosis can be even further increased based on maternal factors that place patients at risk of thrombosis, including advanced maternal age, African American race, some complications of pregnancy and childbirth, as well as caesarian sections.

While all women undergo these physiologic changes, not all develop ischemic heart disease and subsequent myocardial infarction. Most cases of PAMI due to IHD related to the hypercoagulable state of pregnancy are associated with the presence of other mechanisms associated with AMI, significant past medical history, or other traditional risk factors [1,4-6]. This report will briefly discuss the pathogenesis of other significant risk factors as seen in this case.

The presence of COVID-19 is an important risk factor to consider as the disease itself has been associated with both venous and arterial thrombotic complications and has been determined to be an independent risk factor for acute MI in patients [8]. The exact pathogenesis of the prothrombotic state associated with COVID-19 patients remains unclear; however, there have been several mechanisms discussed in recent literature [9]. Proposed mechanisms have been related to the activation of the coagulation cascade in the setting of endothelial injury and inflammation, as well as an increase and amplification of proinflammatory cytokine activation causing a cytokine storm, which in turn promotes a prothrombotic state [9]. Studies have shown COVID-19 as an independent risk factor for acute MI in patients, making acute infection an important factor to consider in this case.

The use of tobacco has long been studied for its correlation with cardiovascular disease and acute MI. Tobacco use places patients of all ages at triple the risk for acute MI with younger patients under the age of 50 having the worst discrepancy in risk with an eight-fold increase as compared to former or never smokers [10,11]. The pathophysiology of cigarette smoking related to cardiovascular disease and acute MI has been linked to the development of atherosclerosis and thrombosis. Tobacco use impairs vasodilatory function, creates inflammation, and modified lipids, which are all integral to the development of atherosclerosis [12]. Tobacco has also been shown to alter the function or concentrations of different components of coagulation, including platelets, antithrombotic factors, prothrombotic factors, and fibrinolytic factors, thus increasing the likelihood of clot formation [12].

Some research has been performed revealing the benefit of placing higher-risk postpartum mothers on preventative anticoagulation. Based on guidelines from the American Society for Hematology (ASH), ACOG recommends prophylactic anticoagulation for patients with a history of VTE or high-risk thrombophilias [5]. However, the evidence to guide clinical decision-making regarding routine pharmacologic thromboprophylaxis postpartum is insufficient, raising the need for more research on this subject. This is a topic that should be studied further as the absolute and relative indications, risk versus benefit, as well as the duration of anticoagulation, remains unclear.

## Conclusions

The physiologic changes associated with pregnancy increase the risk of thrombosis and, subsequently, IHD especially in the presence of additional factors as seen in this case, including advanced maternal age, African American race, obesity, cesarean section, and other medical conditions, i.e. tobacco use or COVID-19. While COVID-19 infection could not have been predicted as a potential risk factor at the time of delivery, the presence of multiple risk factors for postpartum thrombosis brings the use of preventative anti-coagulation into question. Physicians should be mindful of the risk of acute MI and associated complications in postpartum females who are at even higher risk during the current COVID-19 pandemic. Further research is needed on the utility of prophylactic anticoagulation in the postpartum period.
